# Proxy WHO Disability Assessment Schedule 2.0 Is Clinically Useful for Assessing Psychosocial Functioning in Severe Mental Illness

**DOI:** 10.3389/fpsyt.2020.00303

**Published:** 2020-04-15

**Authors:** Anne B. Koopmans, Daphne van Hoeken, Diana E. Clarke, David J. Vinkers, Peter N. van Harten, Hans W. Hoek

**Affiliations:** ^1^Parnassia Academy, Parnassia Psychiatric Institute, The Hague, Netherlands; ^2^School for Mental Health and Neuroscience, Maastricht University, Maastricht, Netherlands; ^3^Division of Research, American Psychiatric Association, Arlington, VA, United States; ^4^Department of Mental Health, Johns Hopkins Bloomberg School of Public Health, Baltimore, MD, United States; ^5^Innova, Psychiatric Centre GGz Centraal, Amersfoort, Netherlands; ^6^Department of Psychiatry, University Medical Center Groningen, University of Groningen, Groningen, Netherlands; ^7^Department of Epidemiology, Mailman School of Public Health, Columbia University, New York, NY, United States

**Keywords:** WHO Disability Assessment Schedule, severe mental illness, functioning, schizophrenia, Caribbean, recovery, rehabilitation

## Abstract

**Aims:**

This study explores how well the World Health Organization Disability Assessment Schedule (WHODAS 2.0) assesses problems with psychosocial functioning in patients with severe mental illness (SMI). Further, we assessed the relationships between psychosocial functioning and psychopathology, medication side effects, treatment setting, and quality of life.

**Methods:**

We performed an observational, cross-sectional study on the island of Curaçao to assess psychosocial functioning in 77 patients with SMI; they mainly had psychotic disorders. We interviewed their healthcare providers using the proxy version of the WHODAS 2.0. In addition, patients were examined for psychiatric symptoms, medication side effects (including drug-induced movement disorders), and quality of life. Associations were examined with Spearman's rank correlation (ρ).

**Results:**

Difficulties in psychosocial functioning were reported by patients with SMI in the WHODAS 2.0 domains of understanding and communicating [mean (M)=34.5, standard deviation (SD)=18.6), participation in society (M=25.5, SD=15.6), and getting along with people (M=24.1, SD=16.1)]. Notably, outpatients had more problems participating in society than inpatients (M=33.6, SD=18.5 *versus* M=23.2, SD=14.1, p=0.03). A positive correlation was observed between drug-induced parkinsonism and the WHODAS 2.0 total score (ρ =0.30; p=0.02), as well as with various subscales, getting around, and household activities.

**Conclusion:**

The proxy version of the WHODAS 2.0 is clinically useful for patients with severe mental illness. The highest scores on the WHODAS 2.0 were found in domains related to interactions with other people and to participation in society. Inpatient status appeared to aid participation in society; this might be due to living in the sheltered clinic environment and its associated daily activities. We further found that drug-induced parkinsonism was associated with a broad spectrum of psychosocial disabilities.

**Clinical Trial Registration:**

ClinicalTrials.gov, identifier: NCT02713672; retrospectively registered in February 2016

## Introduction

The term “severe mental illness” (SMI) was introduced by the USA Substance Abuse and Mental Health Services Administration to differentiate between psychiatric patients with and without problems in daily functioning. Criteria for SMI include excessive disturbance and clinically significant distress or impairment in social, occupational, or other important areas of functioning. Although, potentially, all Diagnostic and Statistical Manual of Mental Disorders, 5th Edition (DSM-5), diagnoses can be involved, most of the patients who qualify as severely mentally ill have been diagnosed with schizophrenia, or another psychotic or major mood disorder. The prevalence of severe mental illness in the USA is about 5% in adults (1).

In the aim of treatment of patients with SMI, there is currently a shift in focus from care to recovery. Recovery is a process in which the most important goal is to regain a meaningful life in the community, including participation in valued activities and fulfillment of social roles (2–5). Participation in everyday activities, such as occupational or educational activities, coupled with adequate social functioning, are considered important indicators of recovery (3, 6). Central to the monitoring of the recovery process is the measurement of patients' psychosocial functioning.

In DSM-5, the World Health Organization Disability Assessment Schedule (WHODAS 2.0) was introduced as a better measure of psychosocial functioning in individuals with psychiatric disorders (7) than the earlier Global Assessment of Functioning (GAF). The WHODAS 2.0 was explicitly designed to exclude items on pathology to avoid confounding symptoms and functioning, which is a core problem with the GAF (8).

Impaired psychosocial functioning in patients with SMI is multifactorial: adverse medication reactions (parkinsonism, obesity), and treatment setting (in- or outpatient) may influence functional recovery (9–13). For example, the medication side effect parkinsonism is associated with poor vocational performance (11), while some studies show positive results for community-based rehabilitation *versus* in-hospital treatment in patients with SMI (12–14).

The WHODAS 2.0 offered valuable insight into patients' experiences, had acceptable validity and psychometric properties, but it was challenging to use with patients with long-term psychotic disorders and several limitations were reported (15, 16), e.g., caregivers generally reported worse functioning than patients themselves. A lack of insight (i.e., awareness of their illness and its consequences) in patients with SMI meant they registered fewer difficulties than expected in the questionnaire (15–17). Researchers also had to try hard to keep such patients focused on answering the questions, possibly because they had cognitive deficits (15). Using the proxy version of the WHODAS 2.0 could parry these limitations, since the interview is done with the patient's healthcare professional or a family member. Yet, to our knowledge, there are only a few publications about using it for patients with severe (neuro)psychiatric disorders (17–19). One study, in Ethiopia in patients with SMI, found that the proxy version had acceptable validity and psychometric properties, but higher mean values and better responsiveness to change than the patient interview version (17).

Our study explored the use of the proxy version of the WHODAS 2.0 for assessing psychosocial functioning in a population with SMI. Further, we assessed the relationships between functioning and psychopathology, medication side effects, treatment setting, and quality of life.

## Methods

Our study was performed in the well-defined geographical area of Curaçao, an island in the Caribbean and part of the former Dutch Antilles. The study was also part of a clinical trial on CYP2D6 and CYP2C19 genotyping in patients with SMI (20). Our study population of 77 patients with SMI was part of the Curaçao Extrapyramidal Syndromes studies cohort (I-XIII) (21, 22). AK, DV, PH, and HH have worked as medical doctors on Curaçao.

Our participants were inpatients recruited in November–December 2014 from the Klinika Capriles (the only psychiatric hospital on the island) and from the psychiatric ward of the prison, and outpatients recruited from the psychiatric outpatient clinic (Psychiaters Maatschap Antillen).

Inclusion criteria for the study were: 1) Antillean ethnicity, defined according to the Dutch Central Bureau of Statistics as born on the former Netherlands Antilles, with at least one parent also born on the former Netherlands Antilles; 2) aged 18 years or older, and 3) meeting SMI criteria, defined as having a mental disorder causing functional impairment as shown by the need for intensive psychiatric care in or out of hospital. Exclusion criteria were: 1) refusal to give written informed consent, or 2) no family member or healthcare professional available and willing to participate in the study.

We approached 86 patients, of whom 77 (90%; 30 females, 47 males) met our inclusion criteria. Eight patients were excluded because they had no healthcare professional or family member available for interview; one patient died before inclusion. After being informed about the study procedures, all patients signed an informed consent form.

Of the 77 proxy questionnaires, two were completed by family members and 75 by healthcare professionals. The healthcare professionals were all nurses, with patient contact at least once a week.

### Measurements

Each patient had a thorough assessment of psychopathology, subjective experience, extrapyramidal symptoms, quality of life, and global functioning. Demographic information was registered. In line with a previous study on SMI patients on Curaçao (21), diagnosis was based on clinical diagnoses as registered by the treating psychiatrist and documented in the participating patients' health records. The healthcare system on Curaçao meets Western standards and the majority of medical specialists are trained in the Netherlands. Information on psychiatric and somatic medication was derived from the electronic patient file. Until recently, Dutch was the only official language on Curaçao and we were able to communicate in Dutch with all healthcare professionals and family members. However, not all our study participants had completed secondary school education, and for those who did not understand Dutch (32 out of 77 patients), we communicated in the local language, Papiamentu, with the help of a bilingual hospital resident-in-psychiatry. To prevent outcome effects because of inter-rater variability, all measurements were done by the same investigator (AK). To prevent halo effects, the nurses were interviewed for the WHODAS with the interviewer blinded to the patient concerned. In this way we could ensure that there was no clinical prejudice in the way the interviewer scored the WHODAS items.

### Functioning

Functioning was measured using the official Dutch translation of the proxy interview version of the WHODAS 2.0 (32 items), which was used with well-informed healthcare professionals or family members (23). Ideally we would also have used the patient version of the WHODAS 2.0 to provide information on the relationship between the two versions of the scale. This information would have helped to strengthen the rationale to use the proxy version of the scale. Previous studies have reported that patients with schizophrenia and schizoaffective disorders are poor raters of their level of functioning but good raters of other characteristics such as quality of life (24–26). Higher levels of depressive symptoms are associated with underestimations in self-reports, while delusions and suspiciousness are associated with overestimation of functioning compared to interviewer judgments (27, 28). In addition, co-authors DH and HH cooperated in the World Health Organization's (WHO) development of the WHODAS 2.0 and concluded that the patient version was difficult to administer to patients with schizophrenia (29). Also, in a small pilot study, first author AK tested this questionnaire. She found that concentration problems hampered the completion of the WHODAS 2.0 questionnaire, possibly due to its length (36 items) and the complex wording of some of the items. These concentration problems negatively affected the reliability of the answers and prompted our use of the informant version. Furthermore, almost none of the patients in the pilot and current study had completed secondary school, which may also explain the difficulties observed in the pilot patients when completing the WHODAS 2.0. Their low level of education may have resulted in problems in answering the complex questions. These observations supported our use of the informant version.

The questionnaire measures patients' functioning in six domains: 1) understanding and communicating (six items), 2) getting around (five items), 3) self-care (four items), 4) getting along with people (five items), 5) work and household activities (each four items), and 6) participation in society (eight items). Four items on work were not included in our study because almost none of the participants had a job—this is in line with the instructions in the Manual for WHO Disability Assessment Schedule WHODAS 2.0 (30).

Standardized summary scores range from 0 to 100, with higher scores indicating greater levels of disability in functioning (30).

The patient interview version of WHODAS has a robust factor structure that is replicated across countries and populations, has high internal consistency (Cronbach's α coefficient of 0.96 for its 36 items, good test-retest reliability (intraclass correlation coefficient of 0.98), good concurrent validity with other quality of life and handicap scales (correlation coefficients 0.45–0.65), and a reasonable sensitivity to change (effect sizes 0.44–1.38) (31, 32). In the general population 90% has a total score of 35 or lower, indicating little or no disability in functioning (30). The proxy interview version shows good internal consistency (0.82–0.99) and good convergent validity in patients with severe mental disorders (17). It has a moderate sensitivity to change (0.14–0.57), which is nonetheless more sensitive than the patient version in this specific (SMI) population (0.17–0.35) (17).

The interviewer, a resident-in-psychiatry (AK), was trained by experts (DH and HH) in scoring the WHODAS 2.0.

### Symptom Severity and Side Effects

The severity of patients' psychopathology was assessed with the Brief Psychiatric Rating Scale (BPRS; 24 items). This rating scale has five subscales: affect (anxiety, guilt, depression, somatic); positive symptoms (thought content, conceptual disorganization, hallucinatory behavior, grandiosity); negative symptoms (blunted affect, emotional withdrawal, motor retardation); resistance (hostility, uncooperativeness, suspiciousness); and activation (excitement, tension, mannerisms–posturing) (33–35). Extrapyramidal side effects were assessed with the St. Hans Rating Scale (SHRS), a multidimensional rating scale for the evaluation of dyskinesia (18 items), parkinsonism (10 items), and dystonia (2 items) induced by antipsychotics. The dyskinesia is scored in two situations: in passive and active circumstances (36). Akathisia, a movement disorder and medication side effect, was measured using the Barnes Akathisia Rating Scale for drug-induced akathisia (BARS; three items) (37, 38).

For all the above scales, higher scores indicate more severe symptoms. AK, a resident-in-psychiatry, was the interviewer for all the scales; she was trained by experts in scoring the SHRS and BARS (PH and HH), and this included a training course with 10 Antillean patients with SMI on Curaçao.

Patient experience of psychopharmacological medication side effects was measured with the Subjective Well-Being Under Neuroleptics Scale (SWN-20; 20 items). This is a self-rating scale that assesses subjective experience over the preceding seven days (39). For patients who were unable to read Dutch, the SWN-20 questions were translated into Papiamentu. Scores of negative statements were recoded to indicate that higher scores on all statements reflected higher levels of well-being. Drug-induced weight gain was measured using body mass index (BMI).

### Quality of Life

Quality of life was assessed with the EQol 5D (EQ-5D; five items), a widely-used scale that measures health status on five dimensions: 1) mobility, 2) self-care, 3) daily activities, 4) pain/discomfort, and 5) anxiety/depression (40, 41). Higher scores indicate a lower quality of life. The EQ-5D shows some overlap with several WHODAS 2.0 domains, including getting around, self-care, and work and household activities.

### Statistics

All analyses were performed with IBM SPSS Statistics Version 25. Normality of the data distribution for all measurements was assessed by inspecting Q-Q plots. We conducted multiple regression analyses to identify how much age and gender contributed independently to the WHODAS 2.0 outcomes. The Mann-Whitney U test was used to compare outcomes between in- and outpatients. Correlations between the WHODAS 2.0 and the other measures were examined using Spearman's rank correlation (ρ), with the significance level set at p < 0.05.

## Results

### Patient Characteristics

Eighteen (23.4%) patients were outpatients and 59 (76.6%) were inpatients. The inpatients lived in housing on the clinic site and had been there for at least 2.5 years. Of those included in the study, 95% had a diagnosis of a psychotic disorder. Other diagnoses included depression, bipolar disorder, substance abuse, and intellectual disability. All patients were using antipsychotic medication [of these, 40 (52%) used biperidene and 45 (58%) were prescribed two types of antipsychotics]. Antidepressant medication was prescribed for 7 (9%) of the patients, while 8 (10%) were using anti-epileptic medication and 33 (42%) were using cardiovascular medication.

### Distribution of Total and Domain Scores of Functioning

The WHODAS 2.0 mean standardized domain and total scores are shown in **Table 1**. The mean total score of the 32-item WHODAS 2.0 scale was 31.2 (SD=16.5) with a minimum score of 2.5 and a maximum score of 78.9. Twenty percent of patients had a total score ≥ 35, which falls above the 90^th^ percentile of overall disability in functioning according to general population data for the WHODAS 2.0 interview version (30).

**Table 1:** Patient characteristics and mean standardized WHO Disability Assessment Schedule (WHODAS) 2.0 scores of in- and outpatients with severe mental illness.

Measurements	Total				Inpatients				Outpatients				Mann Whitney-U	
	N	Mean	SD		N	Mean	SD		N	Mean	SD		U	p-value
Age in years		51.4	11.4			52.4				48.2			404.5	.128
Female (%)		38.9				39.0				38.9				.994^#^
WHODAS 2.0 domains														
Understanding and communicating	77	34.5	18.6		59	35.3	18.0		18	31.9	20.8		470	.46
Getting around	77	14.1	18.6		59	13.2	18.1		18	17.0	20.4		465	.41
Self-care	77	9.0	11.1		59	8.9	11.5		18	9.0	10.1		510	.79
Getting along with people	69	24.1	16.1		55	23.4	16.2		14	26.8	16.2		327.5	.39
Household activities	75	18.5	21.0		59	17.1	20.6		16	23.8	22.4		389.5	.27
Participation in society	74	25.5	15.6		58	23.2	14.1		16	33.6	18.5		**292.5**	.02*
Total score^†^	66	31.2	16.5		54	29.6	15.5		12	38.3	19.6		223.0	.09

SMI, severe mental illness; WHODAS, World Health Organization Disability Assessment Schedule, ^†^ standardized total score of the 32-item scale (exclusion of work items), ^#^chi square, * significant at the.05 level, N number of patients, SD standard deviation

Multiple regression analyses showed no difference in the distribution of WHODAS 2.0 scores according to age or gender.

Re-analysis of the outcomes, excluding the two questionnaires filled out by family members, did not change the results.

### Functioning and Psychopathology

The correlations between psychopathological symptoms as measured by the BPRS and WHODAS 2.0 scores are shown in **Table 2**.

**Table 2:** Correlations (Spearman rho) between scores on WHO Disability Assessment Schedule (WHODAS) 2.0 and other measures in patients with severe mental illness.

	WHODAS 2.0 domains						
Measurements	Understanding and communicating	Getting around	Self-care	Getting along with people	Household activities	Participation in society	Total score^†^
Number of patients	77	77	77	69	75	74	66
BPRS: 24 items (1–7)							
Affective symptoms	.19	.21	.14	.06	.16	.23	.21
Positive symptoms	.14	.05	.16	−.12	.24	.10	.14
Negative symptoms	.28*	.24*	.27*	.10	.14	.09	.29*
Resistance	.09	.01	.01	.21	.04	.28*	.18
Activation	.02	−.19	−.07	.01	−.19	.10	−.05
Total score	29*	.21	.23	.06	.20	.35**	.34**
SHRS: 30 items (0–6)							
Dyskinesia	.15	−.13	.02	−.22	−.06	−.16	−.13
Parkinsonism	.24*	.37**	.16	.10	.23*	.11	.30*
Dystonia	.05	.01	−.18	.02	.10	.11	.05
Acathisia	.02	−.07	−.13	.01	−.12	.12	−.04
BARS: 3 items (0–3)	.01	−.09	−.14	.01	−.11	.12	−.06
SWN-20: 20 items (1–6)	−.27*	−.13	−.08	−.11	−.10	−.22	−.24
BMI	−.16	.07	.05	−.03	−.12	−.02	−.08
EQ-5D: 5 items (1–3)	.22	.39**	.10	−.01	.20	.26*	.33*

SMI, severe mental illness; WHODAS, World Health Organization Disability Assessment Schedule; BPRS, Brief Psychiatric Rating Scale; SHRS, St. Hans Rating Scale; BARS, Barnes Akathisia Rating Scale for drug-induced akathisia; EQ-5D, EQol 5-D; SWN-20, Subjective Well-Being Under Neuroleptics Scale; BMI, body mass index, ^†^standardized total score of the 32 item scale (exclusion of work items), * significant at.05 level, ** significant at.01 level

### Functioning and Medication Side Effects

The correlations between SHRS, BARS, SWN-20, and BMI scores with WHODAS 2.0 scores are shown in **Table 2**. The results indicate that a higher level of drug-induced parkinsonism was associated with more problems in functioning in the areas understanding and communicating, getting around, and household activities.

Higher well-being under neuroleptics is associated with better functioning in the domain of understanding and communicating.

### Functioning and Quality of Life

The correlations between the EQ-5D and WHODAS 2.0 scores are shown in **Table 2**. A higher WHODAS 2.0 score (worse global functioning) is associated with a higher EQ-5D score (lower quality of life).

## Discussion

This study is one of the first to examine WHODAS 2.0 proxy scores of psychosocial functioning in patients with SMI. Our results show that symptom severity and medication side effects have a negative association with functioning, quality of life, and subjective well-being in patients with SMI. They also recorded the highest scores on WHODAS 2.0 domains related to interactions with other people and participating in society, revealing their greater psychosocial disabilities in these areas. These results are consistent with earlier findings, in which psychotic patients reported problems with community activities and experienced barriers in their environment (15).

### Psychopathology

The correlation between psychopathology symptoms and participation in society confirms earlier findings in SMI populations, in which patients with more severe psychopathology reported more difficulties in functioning on the WHODAS 2.0 (12- and 36-item versions), whereas patients with fewer mental health problems reported better scores for recovery (17, 18, 42). We did not find a significant correlation between psychosocial functioning and positive symptoms; this is in agreement with an earlier study in patients with schizophrenia, which found no relationship between positive symptoms and social network size (43). It suggests that the primary focus of traditional treatment on symptoms like hallucinations and thought disorders is not necessarily associated with better functioning.

### Treatment Setting

In contrast to our expectations, outpatients reported more problems with participation in society than inpatients. This contrasts with results from a previous validation study of patients with schizophrenia, in which outpatients demonstrated better social functioning than inpatients, as rated by independent assessors (44). There are several possible explanations: first, inpatients may have better participatory skills than outpatients. This seems unlikely however, because their psychopathology symptoms did not differ. Another explanation stems from healthcare professionals perhaps having different frames of reference for in- and outpatients and their participation in society. For outpatients they may have other insights into barriers for functioning (e.g., stigmatization) in society. It is possible that long- and short-term inpatients and their healthcare professionals are accustomed to the status quo and have lower expectations of life than outpatients (and their healthcare professionals) who are constantly confronted with their disabilities when trying to operate in society.

The third, and in our opinion the most plausible, explanation is that participation is easier in the sheltered environment of an inpatient setting, in which co-citizens also have severe mental disorders, and where social activities are organized, patients are encouraged to participate in activities, basic life needs are taken care of, and barriers and problems are partly resolved by caregivers.

### Medication Side Effects

There was a significant correlation between drug-induced parkinsonism and problems in getting around and performing household activities. This could be related to the symptoms of drug-induced parkinsonism such as tremor, bradykinesia, rigidity, and postural instability (45). These motor symptoms can directly influence walking pattern and daily household tasks (46, 47). In addition, there was a significant correlation between parkinsonism and understanding and communicating. This is in line with earlier reports about the non-motor signs of drug-induced parkinsonism e.g., cognitive dysfunction, apathy, and mood disorders (48–50). Hence, drug-induced parkinsonism appears to be associated with a broad spectrum of dysfunction and may be a barrier to recovery.

### Strengths and Limitations

We studied a difficult-to-examine population of severely ill psychiatric patients who were recruited from all three psychiatric institutions on Curaçao. There was no selection regarding the type of psychiatric care or medication, presence of side effects, or treatment response when the participants were recruited. This resulted in a group of patients who are representative of the general population with SMI on Curaçao. Although nearly half of our participants were unable to communicate in Dutch, we were able to use the official Dutch translation of the WHODAS 2.0, as all the healthcare professionals are educated in Dutch. So far, no local-language versions of the WHODAS 2.0 are available for populations in the Caribbean who do not speak English or Dutch. Although it would have been valuable to interview a relative, most of the patients did not have an engaged relative. A personal healthcare provider who was engaged with all the patient's personal activities was interviewed instead. In a previous study assessing patient functioning, it was found that high-contact clinicians generated ratings of everyday functioning that were more closely linked to patients' ability scores than those from friends or relatives (51).

Not all patients were able to read the Dutch language. To be able to administer the SWN-20 self-report questionnaire to these patients, it was translated by a bilingual (Papiamentu-Dutch) resident-in-psychiatry. No back translation was performed and we did not formally ensure whether the validity and reliability of the questionnaire were still intact. Failure to complete back translation of the SWN-20 might have affected the reliability and validity of the data from the questionnaire and its subsequent correlation with the WHODAS 2.0 and its domains. Our examination of the correlation between the WHODAS 2.0 and its domains and the SWN-20 in the subset of patients who completed the Dutch version of the scale (n=38) showed similar results to those on the subset that completed the translated version of the SWN-20 (n=39). This indicates that the negative impact of the failure to back translate was minimal.

Problems with using questionnaires on functioning with patients with poor insight and cognitive deficits, as reported in earlier studies and confirmed in a pilot study, were parried by using the proxy-version with well-informed third parties. A limitation of this study is that we did not interview the patients themselves using the WHODAS 2.0, because a pilot study with the patient version of the questionnaire showed that patients' concentration problems negatively affected the reliability of their answers. Therefore we could not compare the information given by patients with that from their proxies.

Responder bias (judgment differences between healthcare professionals attending inpatients *vs.* outpatients) could not be ruled out, however, the results indicate that useful information on psychosocial functioning can be retrieved from proxies.

Lastly, both the small sample size and the non-normal distribution of our data, requiring non-parametric tests, are challenges for the power of our analyses, and thus the strength of our findings. Non-parametric tests (here: Spearman rho and Mann Whitney) have less statistical power than their parametrical equivalents. As our main purpose was to explore relationships between different measures, we chose to show all individual results of our small sample size study. Thus, together with the fact that the study has a cross-sectional design, the results need to be treated with caution. Future studies, using larger sample sizes are needed to confirm our findings.

## Conclusions

In this exploratory study, we show that the proxy version of the WHODAS 2.0 is a clinically useful instrument for patients with severe mental illness and that it provides good insight into the psychosocial functioning of these patients. In patients with lack of insight into their disease or severe cognitive deficits, this proxy questionnaire version can be used to determine useful information about patients' problems in psychosocial functioning. The highest scores on the WHODAS 2.0 (i.e., indicating the greatest difficulties in functioning) were found in domains related to interactions with other people and participation in society. Inpatients were reported to experience fewer problems with participation in society than outpatients, which could be due to the adapted circumstances and sheltered environment of the clinic. We found drug-induced parkinsonism is associated with a broad spectrum of social disabilities. Before any practice or policy recommendations can be made, our results need to be replicated, preferably with larger samples. Wherever possible, researchers should include the patient interview version of the WHODAS 2.0 in order to compare the outcomes of the two versions of the questionnaire.

## Data Availability Statement

The raw data supporting the conclusions of this article will be made available by the authors, without undue reservation, to any qualified researcher.

## Ethics Statement

The study protocol was approved by the Institutional Review Board of Maastricht University, the Netherlands. The procedures were in accordance with the ethical standards of the Helsinki Declaration of 1975 (as revised in 1983). All patients signed written informed consent forms after being informed about the study procedures. The study was registered in an international trial registry at http://www.clinicaltrials.gov (*NCT02713672*).

## Author Contributions

Study conception and design: AK, DV, DH, DC, PH, HH. Acquisition of data: AK. Analysis and interpretation of data: AK, DV, DC, DH. Drafting of manuscript: AK. Critical revision: AK, DC, DH, DV, PH, HH. All authors read and approved the manuscript.

## Funding

This study was funded by the Netherlands Organization for Health Research and Development (ZonMW, Protocol ID 70-72600-98-005). ZonMW provided financial support only and had no role in the analysis or interpretation of the data, nor in the preparation, review, or approval of the manuscript.

## Conflict of Interest

The authors declare that the research was conducted in the absence of any commercial or financial relationships that could be construed as a potential conflict of interest.
